# Group of longitudinal adverse event patterns after the fourth dose of COVID-19 vaccination with a latent class analysis

**DOI:** 10.3389/fpubh.2024.1406315

**Published:** 2024-07-30

**Authors:** Chika Yamamoto, Yurie Kobashi, Takeshi Kawamura, Yoshitaka Nishikawa, Hiroaki Saito, Fumiya Oguro, Tianchen Zhao, Morihito Takita, Toyoaki Sawano, Akihiko Ozaki, Toshiki Abe, Naomi Ito, Yudai Kaneko, Aya Nakayama, Masatoshi Wakui, Tatsuhiko Kodama, Masaharu Tsubokura

**Affiliations:** ^1^Department of Radiation Health Management, Fukushima Medical University School of Medicine, Fukushima City, Fukushima, Japan; ^2^Department of General Internal Medicine, Hirata Central Hospital, Hirata, Fukushima, Japan; ^3^Proteomics Laboratory, Isotope Science Center, The University of Tokyo, Tokyo, Japan; ^4^Laboratory for Systems Biology and Medicine, Research Center for Advanced Science and Technology, The University of Tokyo, Tokyo, Japan; ^5^Department of Internal Medicine, Soma Central Hospital, Soma, Fukushima, Japan; ^6^Department of Surgery, Jyoban Hospital, Iwaki, Fukushima, Japan; ^7^Department of Breast and Thyroid Surgery, Jyoban Hospital, Iwaki, Fukushima, Japan; ^8^Medical & Biological Laboratories Co., Ltd, Minato-ku, Tokyo, Japan; ^9^Department of Laboratory Medicine, Keio University School of Medicine, Shinjuku-ku, Tokyo, Japan; ^10^Minamisoma Municipal General Hospital, Minamisoma, Fukushima, Japan

**Keywords:** COVID-19, vaccination, adverse events, latent class analysis, Fukushima cohort

## Abstract

**Introduction:**

Vaccination has been implemented as a useful measure to combat the COVID-19 pandemic. However, there is a tendency for individuals to avoid vaccination due to the possibility of adverse events, making it important to investigate the relationship between COVID-19 vaccines and their adverse events. This study explored longitudinal adverse event patterns and factors that influence adverse events following the second to fourth doses of the COVID-19 vaccine through a latent class analysis.

**Methods:**

Participants were recruited from the Fukushima Prefecture and included individuals who had completed four doses of the COVID-19 mRNA vaccine. This study utilized data from questionnaire surveys and blood collection conducted between September 2021 and November 2022. In the questionnaire, factors such as sex, age, medical history, medication, type of vaccine administered, and adverse events following vaccination were recorded. Additionally, in the blood data, serological tests [IgG(S)] and cellular immune responses (T-spot) were measured. Descriptive statistics, latent class analysis, multivariable logistic regression, and multiple regression analyses were performed to identify the longitudinal adverse event patterns and influencing factors. By analyzing adverse events over time, we identified two distinct groups: those less prone to experiencing adverse events (Group 1) and those more susceptible (Group 2) to latent class analysis.

**Results:**

A total of 1,175 participants were included after excluding those without any adverse events. The median age of the participants in Group 1 was 70 years, and in Group 2 it was 51 years. The proportion of female participants was 298 in Group 1 and 353 in Group 2. Patients in Group 2 were significantly younger (*p* < 0.001) and more likely to be female (*p* < 0.001) than those in Group 1. Furthermore, the median IgG(S) value after the fourth vaccination was 3,233 AU/mL in Group 1 and 4,059.39 AU/mL in Group 2. The median T-spot value was 15.4 in Group 1 and 28.5 in Group 2. Group 2 showed significantly higher IgG(S) and T-spot values after the fourth vaccination (*p* < 0.001).

**Discussion:**

Our findings suggest that factors other than age, particularly sex and a history of allergies, significantly influence the likelihood of experiencing adverse events. Groups categorized by latent class analysis for longitudinal adverse events are expected to be valuable for optimizing vaccination strategies and formulating public health measures.

## 1 Introduction

The COVID-19 pandemic has significantly impacted global health, causing serious health issues worldwide. Vaccination is one of the most critical measures implemented in response to these challenges. Vaccination is expected to facilitate antibody acquisition and reduce the severity of infection symptoms. However, concerns regarding the safety of vaccines (1–3) and their adverse events (4–7) have led to vaccine hesitancy (8–11). Previous studies have reported a tendency for individuals who experience more adverse events to avoid vaccinations (2). Therefore, it is important to investigate the relationship between COVID-19 vaccines and adverse events.

Various studies have addressed the adverse events associated with COVID-19 vaccines (12–15). For instance, mass vaccinations have been implemented in many countries, and observations of adverse events that result from these vaccinations have sparked discussions about their efficacy (16, 17) and safety. According to a report by Urakawa et al. (18), factors such as young age, female sex, and the absence of comorbidities have been identified to influence adverse events. However, within the same cohort, there is limited information on the longitudinal sequence of adverse events following COVID-19 vaccination, existing groups, and factors that influence such reactions.

Continuous testing for COVID-19 has been conducted in areas affected by disasters, particularly in the cities and villages of Hamadori in Fukushima Prefecture, a region impacted by the Great East Japan Earthquake and the Fukushima Daiichi Nuclear Power Plant accident. This involved a cohort study (19–27) that targeted approximately 2,500 individuals, including local government officials, hospital staff, and residents. Following the mass vaccination campaign in Japan, blood samples were collected every 3 months from this cohort to continuously monitor adverse events and antibody levels post-vaccination.

This study aimed to understand the longitudinal characteristics of adverse events to the second to fourth doses of the COVID-19 vaccine. By employing Latent Class Analysis (LCA) to cluster the time series of these reactions, we analyzed adverse events following each vaccination dose. Our findings provide insights into the patterns of adverse events to COVID-19 vaccinations over time.

## 2 Method

### 2.1 Study participants

Study participants were recruited from residents and healthcare workers living in Soma City, Minamisoma City, Hirata Village, and Iwaki City in Fukushima Prefecture. Participation was based on written consent obtained from the participants.

This study was approved by the Ethics Committees of Hirata Central Hospital (Number 2021-0611-1) and Fukushima Medical University (Number 2021-116) and was conducted in accordance with the ethical guidelines of the World Medical Association (Declaration of Helsinki).

The inclusion criteria for this study were as follows: individuals who had completed four doses of COVID-19 mRNA vaccines, including BNT162b2 (Pfizer/BioNTech, New York, USA), mRNA-1273 (Moderna, Cambridge, MA, USA), or bivalent vaccines such as Comirnaty Bivalent Original/Omicron BA.1/BA.2 (Pfizer/BioNTech), Comirnaty Bivalent Original/Omicron BA.4/BA.5 (Pfizer/BioNTech), Spikevax Bivalent Original/Omicron BA.1/BA.2 (Moderna, Cambridge), or Spikevax Bivalent Original/Omicron BA.4/BA.5 (Moderna, Cambridge).

### 2.2 Study design

This is an observational historical cohort study that is part of a broader evaluation of antibody testing following COVID-19 mRNA vaccination in the Fukushima Prefecture. This study utilized data from up to five blood collections and questionnaire surveys conducted between September 2021 and November 2022.

#### 2.2.1 Data collection

The questionnaire survey covered various aspects, including age, sex, weight, height, alcohol consumption habits, smoking habits, medication intake, underlying diseases, types of the second, third, and fourth vaccine doses, adverse events after each vaccine dose, and infection status. Medications included steroids, immunosuppressants, and biologics, whereas underlying diseases included hypertension, diabetes, and hyperlipidemia. The adverse events included localized pain, fever, headache, muscle/joint pain, diarrhea, nausea, and dizziness. These questionnaires were collected on paper. Responses were managed in Microsoft Excel (Microsoft Inc., Redmond, CA, USA) using an ID that excluded personal information, and data quality control was performed by at least two people checking the responses.

#### 2.2.2 Serological assay

In the serological assay, IgG antibodies against the S1 protein [IgG(S)] were measured. The assay was conducted using a chemiluminescent immunoassay at the University of Tokyo, Japan. The reagents used were iFlash 3000 (YHLO Biotech, Shenzhen, China) and iFlash-2019-nCoV series (YHLO Biotech). The cutoff value for each item [IgG(S)] was set at 10 AU/mL according to the official cutoff values prescribed by the manufacturer.

#### 2.2.3 Cellular immune response

The cellular immune response was evaluated using an ELISpot assay with T-spot COVID (Oxford Immunotec, UK). The collected blood samples were sent for measurement on the same day to LSI Medience Corporation (Tokyo, Japan), where the ELISpot assay targeting the spike protein as the antigen was performed. In this assay, effector T cells producing interferon-gamma were counted as spots on the well. The results were compared to those of the positive and negative control wells. The number of spots was assessed according to official guidelines, with a maximum of 50 spots. More than 50 spots were considered as “over 50,” more than seven spots as “reactive,” seven spots as “borderline,” and less than five spots as “non-reactive.”

### 2.3 Statistical analysis

This study aimed to understand the longitudinal characteristics of adverse events related to the second through fourth doses of the COVID-19 vaccine. Therefore, instead of using standard regression analysis with adverse events as dependent variables at each vaccination point, we chose to use LCA to examine the time series data of the same individuals at three points in time.

First, a LCA was conducted on the number of systemic adverse events (fever, fatigue, headache, muscle/joint pain, diarrhea, nausea, and dizziness, as well as menstrual irregularities for females only) following the second to fourth vaccine doses. Based on the results of the LCA of systemic adverse events after the second to fourth vaccine doses, the participants’ characteristics were compared using descriptive statistics (Table 1). Categorical variables (sex, alcohol intake, smoking, medication, underlying diseases, types of vaccines, and adverse events) were summarized as frequencies, and continuous variables (age) were summarized as the median and the interquartile range (IQR). In addition, LCA was used to identify groups of adverse event severity after the second to fourth COVID-19 vaccinations. Entropy (28) was taken into account in the appropriate model by LCA, and the model with the best entropy was selected. Two groups were divided by the appropriate model. Of the groups classified, Group 1 exhibited the fewest systemic adverse events and Group 2 exhibited the most adverse events. Furthermore, multivariate logistic regression analysis was used to elucidate the characteristics of the participants, using Group 2 as a reference. Age, sex, vaccine type, medication, and underlying diseases were included as independent variables. Finally, a multiple regression analysis was used to investigate immunity after the fourth vaccine dose. Log-transformed IgG(S) and T-spot titers were used as outcomes in multivariable analysis. The dependent variables were IgG(S) and T-spot values, and the independent variables included sex, age, types of the 3rd and 4th vaccine doses, Group, the period between the fourth vaccine dose and blood collection, smoking habits, alcohol drinking habits, medication, and underlying diseases.

**Table 1:** Participant characteristics (*N* = 1,175).

	Group 1 (Low adverse event group, *n* = 571) *n* (%)	Group 1_*n*: available numbers	Group 2 (High adverse event group, *n* = 604) *n* (%)	Group 2_*n*: available numbers
Age (year) (median [IQR])	70 [62–80]	571	51 [39–63]	604
Sex Female	298 (54.6)	546	353 (71.2)	496
Vaccination kind of fourth dose				
Pfizer	144 (25.2)	571	128 (21.2)	604
Moderna	427 (74.8)		476 (78.8)	
Smoking habit	75 (13.4)	558	83 (13.9)	597
Alcohol consumption	219 (39.4)	556	249 (41.8)	596
Daily medicine				
Steroid	22 (3.9)	561	8 (1.4)	593
Immunosuppression	11 (2.0)	560	5 (0.8)	594
Biologics	2 (0.4)	558	3 (0.5)	593
Comorbidity				
Hypertension	293 (51.4)	570	148 (24.5)	604
Diabetes	84 (14.7)	570	48 (8.0)	604
Dyslipidemia	109 (19.1)	570	76 (12.6)	604
Adverse event after second dose				
Local pain	226 (39.6)	571	391 (64.7)	604
Over 37.5 degree fever	23 (4.0)		240 (39.7)	
Fatigue	69 (12.1)		437 (72.4)	
Headache	14 (2.5)		246 (40.7)	
Joint pain	55 (9.6)		272 (45.0)	
Adverse event after third dose				
Local pain	274 (48.0)	571	423 (70.0)	604
Over 37.5 degree fever	21 (3.7)		237 (39.2)	
Fatigue	43 (7.5)		413 (68.4)	
Headache	13 (2.3)		256 (42.4)	
Joint pain	39 (6.8)		295 (48.8)	
Adverse event after fourth dose				
Local pain	242 (42.4)	571	439 (72.7)	604
Over 37.5 degree fever	23 (4.0)		280 (47.4)	
Fatigue	51 (8.9)		439 (72.7)	
Headache	15 (2.6)		256 (42.4)	
Joint pain	32 (5.6)		279 (46.2)	
IgG(S) of fourth dose (median [IQR])	3,233.3 [1,380.3–4,221.0]	571	4,059.39 [2,092.5–5,082.7]	604
T-spot of fourth dose (median [IQR])	15.4 [4.0–22.0]	571	28.5 [11.0–50.0]	604

IQR, Interquartile Range.

The statistical analysis was carried out without filling in missing data; therefore, there were different numbers of missing data for each item. The number of available data for each item is indicated in the table as “*n*: available numbers.” All percentages given in this table are of the available values.

All statistical analyses were performed using Stata/BE 17 (TX 77845, USA), and statistical significance was set at *p* < 0.05.

## 3 Results

### 3.1 Participant characteristics

**Figure 1:**
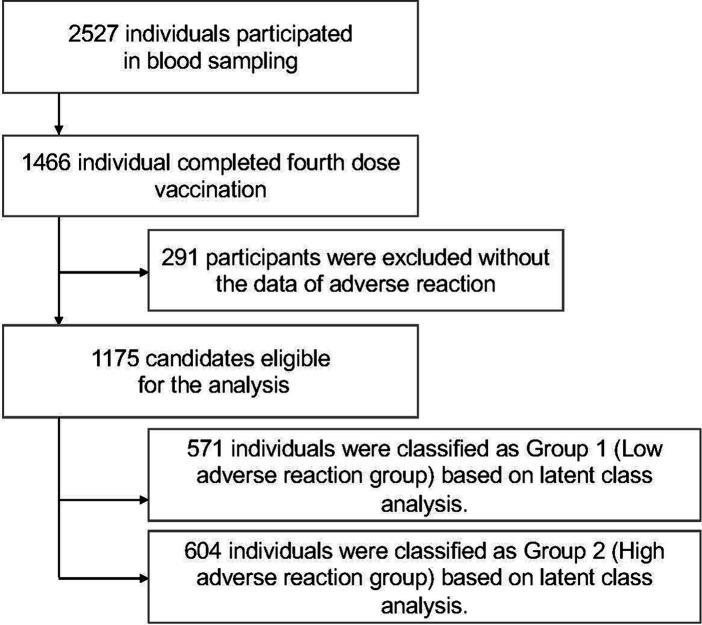
Flowchart of inclusion and exclusion criteria for the study participants. A total of 2,527 subjects from the Fukushima Cohort Study participated in up to five blood draws and answered questionnaires between September 2021 and November 2022; those who received the fourth dose of COVID-19 vaccine (*n* = 1,466) were enrolled and those with no documented adverse effects after the second, third or fourth vaccine doses (*n* = 291) were excluded. A total of 1,175 individuals were ultimately included in the study.

A total of 2,527 subjects participated in up to five blood draws and questionnaires conducted between September 2021 and November 2022. Of these, a total of 1,466 subjects met the criteria for completing the fourth vaccination. Next, 291 subjects with no documented adverse events after the second, third, or fourth vaccination were excluded. Ultimately, a total of 1,175 individuals were included in the study (Figure 1).

The characteristics of the 1,175 study participants, classified into Groups 1 and 2, are summarized in Table 1. The median age of the participants in Group 1 was 70 years [interquartile range (IQR): 62–80], and that in Group 2 was 51 years (IQR: 39–63). The proportion of female participants was 298 (54.58%) in Group 1 and 353 (71.17%) in Group 2. For the fourth COVID-19 vaccination, Pfizer was administered to 144 (25.22%) participants in Group 1 and 128 (21.19%) in Group 2, whereas Moderna was administered to 427 (74.48%) participants in Group 1 and 476 (78.81%) in Group 2. Total adverse events following each vaccine dose were as follows: after the second dose, Group 1 had 387 cases and Group 2 had 1,586 cases; after the third dose, Group 1 had 390 cases and Group 2 had 1,624 cases; and after the fourth dose, Group 1 had 363 cases while Group 2 had 1,693 cases. These figures include instances where a single participant experienced multiple side effects. The number of participants with smoking habits was 75 (13.44%) in Group 1, 83 (13.99%) in Group 2, and the number of participants who consumed alcohol was 219 (39.39%) in Group 1, and 249 (41.78%) in Group 2. The median IgG(S) value after the fourth vaccination was 3,233 AU/mL (IQR: 1,389.3–4,221.0) in Group 1 and 4,059.39 AU/mL (IQR: 2,092.5–5,082.7) in Group 2. The median T-spot *t* value was 15.4 (IQR: 4.0–22.0) in Group 1 and 28.5 (IQR: 11.0–50.0) in Group 2.

### 3.2 Participants classification

**Figure 2:**
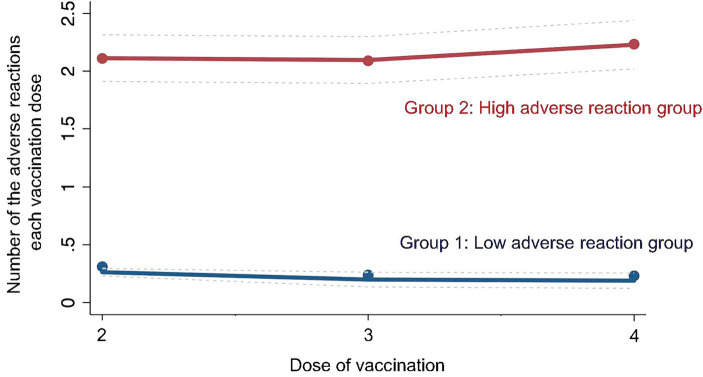
Group of latent class analysis. The figure shows group classification results based on latent class analysis of the study participants’ adverse events.

Participants were analyzed using LCA based on adverse events following the second to fourth COVID-19 vaccinations. The analysis showed that the participants were classified into two groups based on the difference in the frequency of adverse events after the second to fourth vaccinations, and the entropy between these two groups was the highest (entropy = 0.787). Therefore, the two groups were classified into the two groups with the highest entropy values. The group with fewer adverse events following the second to fourth vaccinations (low adverse event group) was designated as Group 1 (*n* = 571), and the group with more frequent adverse events (high adverse event group) was designated as Group 2 (*n* = 604) (Figure 2).

### 3.3 Factors related to systemic adverse events

The results of the mulitvariate logistic regression analysis, with the likelihood of being classified into Group 2 (high adverse event group) as the dependent variable, are shown in Table 2. Participants in Group 2 were significantly younger [Relative Risk Ratio (RRR): 0.93, 95% CI: 0.917–0.938, *p* < 0.001] and more likely to be female (RRR: 2.35, 95% CI: 1.723–3.206, *p* < 0.001) than those in Group 1. No significant associations were found between the type of vaccine administered for the third to fourth doses, intake of steroids, immunosuppressants, biologics, or preexisting conditions such as asthma, rheumatism, antigenic diseases, and immunosuppression. However, a history of allergies was significantly associated with being in Group 2 (RRR: 2.12, 95% CI: 1.064–4.210, *p* = 0.033).

**Table 2:** Multinomial logistics regression analysis for predicting Group 2 (High adverse event group) (*n* = 1,015).

	RRR (95% CI)	*p* value
Age	0.93 (0.917–0.938)	**<0.001**
Sex (female)	2.35 (1.723–3.206)	**<0.001**
Vaccination kind of fourth dose (Moderna)	1.08 (0.762–1.523)	0.68
Vaccination kind of third dose (Moderna)	0.82 (0.590–1.137)	0.23
Daily medicine (yes)		
Steroid	0.34 (0.109–1.087)	0.069
Immunosuppression	0.63 (0.141–2.854)	0.55
Biologics	4.47 (0.420–47.728)	0.22
Comorbidity (yes)		
Asthma	1.62 (0.802–3.257)	0.179
Rheumatism	0.53 (0.155–1.796)	0.31
Antigen Disease	1.06 (0.134–8.437)	0.95
Allergy	2.12 (1.064–4.210)	**0.033**
Immunological Disorder	31,161.37 (0)	0.99

95% CI, 95% confidence interval; RRR, Relative Risk Ratios.

The results of the multinomial logistic regression analysis conducted with Group 1 as the reference group. RRR indicates Relative Risk Ratios in the Multinomial logistics regression analysis. Coefficient (95% CI) is the Coefficient in the Multiple regression analysis, where 95% CI indicates the Confidence Interval.

### 3.4 Impact on IgG(S) after the fourth vaccination

The results of the multiple regression analysis of IgG(S) values after the fourth COVID-19 vaccination, involving 981 participants, are presented in Table 3. The high adverse event group (Group 2) showed a statistically significant positive association with IgG(S) values (coefficient: 0.114, 95% CI: 0.067–0.160, *p* < 0.001). Additionally, a longer interval between the fourth COVID-19 vaccination and the fifth blood sampling was significantly associated with a decrease in IgG(S) values (coefficient: −0.003, 95% CI: −0.004 to −0.002, *p* < 0.001). Age, sex, vaccine type, and smoking habits did not significantly affect IgG(S) values. However, higher alcohol consumption was significantly associated with a decrease in IgG(S) values (coefficient: −0.070, 95% CI: −0.115 to −0.025, *p* = 0.002). Medication intake, including steroids (coefficient: 0.130, 95% CI: −0.004 to 0.265, *p* = 0.058), did not show a significant association with an increase in IgG(S), whereas the use of immunosuppressants (coefficient: −0.270, 95% CI: −0.449 to −0.090, *p* = 0.003) and biologics (coefficient: −0.310, 95% CI: −0.599 to −0.022, *p* = 0.035) was significantly associated with a decrease in IgG(S) values. Furthermore, no significant associations were observed between IgG(S) values and preexisting conditions, such as hypertension, diabetes, or hyperlipidemia.

**Table 3:** Multiple regression analysis for Log transformed IgG(S) after the fourth vaccination dose (*n* = 981).

	Coefficient (95% CI)	*p* value
Group 2 (High adverse event group)	0.114 (0.067–0.160)	**<0.001**
Age	−0.001 (−0.002 to 0.001)	0.36
Sex (female)	−0.017 (−0.064 to 0.029)	0.47
Vaccination kind of fourth dose (Moderna)	0.039 (−0.008 to 0.086)	0.107
Vaccination kind of third dose (Moderna)	0.008 (−0.037 to 0.054)	0.73
Interval date between the fourth vaccination dose and fifth blood sampling	−0.003 (−0.004 to 0.002)	**<0.001**
Smoking habit	−0.028 (−0.088 to 0.032)	0.36
Alcohol consumption	−0.070 (−0.115 to 0.025)	0.002
Daily medicine		
Steroid	0.130 (−0.004 to 0.265)	0.058
Immunosuppression	−0.270 (−0.449 to 0.090)	**0.003**
Biologics	−0.310 (−0.599 to 0.022)	**0.035**
Comorbidity		
Hypertension	−0.007 (−0.054 to 0.040)	0.77
Diabetes	−0.045 (−0.107 to 0.017)	0.157
Dyslipidemia	0.003 (−0.051 to 0.057)	0.91

95% CI, 95% confidence interval. RRR indicates Relative Risk Ratios in the Multinomial logistics regression analysis. Coefficient (95% CI) is the Coefficient in the Multiple regression analysis, where 95% CI indicates the Confidence Interval.

### 3.5 T-spot values after the fourth vaccination

The results of the multiple regression analysis of the log-transformed values of T-spots after the fourth COVID-19 vaccination are presented in Table 4. Participants in the high adverse event group (Group 2) showed a significant increase in T-spot values (coefficient: 0.193, 95% CI: 0.120–0.267, *p* < 0.001), while age was significantly associated with a decrease in T-spot values (coefficient: −0.008, 95% CI: −0.010 to −0.005, *p* < 0.001). Participants who received the fourth dose had higher T-spot values (coefficient:0.098, 95% CI: 0.021–0.176, *p* = 0.013); conversely, smoking habits were associated with a decrease in T-spot values (coefficient: −0.123, 95% CI: −0.217 to −0.028, *p* = 0.011). Furthermore, participants taking immunosuppressants showed a significant decrease in T-spot values (coefficient: −0.275, 95% CI: −0.546 to −0.004, *p* = 0.046).

**Table 4:** Multiple regression analysis for Log transformed T-spot(S) after the fourth vaccination dose (*n* = 809).

	Coefficient (95% CI)	*p* value
Group 2 (High adverse event group)	0.193 (0.120–0.267)	**<0.001**
Age	−0.008 (−0.010 to 0.005)	**<0.001**
Sex (female)	0.0004 (−0.076 to 0.075)	0.99
Vaccination kind of fourth dose (Moderna)	0.098 (0.021–0.176)	**0.013**
Vaccination kind of third dose (Moderna)	0.064 (−0.009 to 0.138)	0.085
Interval date between fourth vaccination dose and fifth blood sampling	−0.001 (−0.002 to 0.001)	0.34
Smoking habit	−0.123 (−0.217 to 0.028)	**0.011**
Alcohol consumption	0.004 (−0.066 to 0.075)	0.90
Daily medicine		
Steroid	0.044 (−0.156 to 0.244)	0.66
Immunosuppression	−0.275 (−0.546 to 0.004)	**0.046**
Biologics	0.103 (−0.316 to 0.522)	0.63
Comorbidity		
Hypertension	−0.004 (−0.078 to 0.070)	0.92
Diabetes	0.003 (−0.100 to 0.106)	0.96
Dyslipidemia	0.043 (−0.047 to 0.133)	0.35

95% CI, 95% confidence interval. RRR indicates Relative Risk Ratios in the Multinomial logistics regression analysis. Coefficient (95% CI) is the Coefficient in the Multiple regression analysis, where 95% CI indicates the Confidence Interval.

## 4 Discussion

This study aimed to understand the longitudinal characteristics of adverse events to the second to fourth doses of the COVID-19 vaccine. By clustering the time series of these reactions using LCA, we identified two distinct groups: one more prone to adverse events and the other less prone to adverse events following COVID-19 vaccination.

The patterns of adverse events over time suggest that factors other than age influence their occurrence. It became clear that there was polarization in the continuation of adverse events over time after vaccination. To the best of our knowledge, previous studies have not extensively explored the persistence of adverse events in post-vaccination time series. Future research to elucidate these factors is crucial for assessing the safety and efficacy of the ongoing vaccination efforts. The existence of groups with consistently high or low risk of adverse events also suggests the need for individualized approaches (29) to vaccine risk management.

Individuals more prone to adverse events included those who were female, were younger, and had a history of allergies. This trend indicates that sex and age may influence immune responses to vaccines. One of the factors that make women more prone to adverse events after vaccination is reactogenicity (5). Investigations into acute COVID-19 (30) (the so-called “post COVID conditions,” or simply “long COVID”) have reported that women (31, 32) are more likely than men to develop adverse events. Furthermore, reports align with findings that the risk of adverse events is higher in women (33–37) and younger (18, 38) individuals after vaccination.

Additionally, the association between a history of allergies (39–41) and the occurrence of adverse events has also been noted. In addition to explaining the potential adverse events to the vaccine, it may be necessary to provide a more detailed explanation regarding the management of these events, especially for patients with a history of allergies. Furthermore, careful observation during administration and consideration of easy access to medical facilities in the event of symptoms are required for follow-up.

In terms of serological outcomes, the group with more adverse events (Group 2) showed higher values in the IgG and T-spot tests. This group was significantly associated with IgG levels, suggesting a correlation between post-vaccination adverse events and antibody levels. The group with consistently high adverse events had higher values in both IgG(S) and T-spot tests. This finding is consistent with that of previous studies (42) that have shown a significant association between systemic adverse events and IgG(S). These findings suggest a potential link between immune responses and adverse events following vaccination (26, 43). Subjects in Group 2, compared to those in Group 1, were significantly younger and predominantly female, allowing for the examination of the relationship between these factors and immune responses. Numerous reports have indicated gender differences in immune response, driven by sex hormones (44, 45) such as testosterone in men and estrogen and progesterone in women, as well as genes derived from sex chromosomes. These hormones, the receptors for which are also found on immune cells, play a crucial role in regulating the immune system (45). For example, estrogen can regulate the production of inflammatory cytokines (46), increase the accumulation of neutrophils, thus promoting an adaptive T-cell response, enhancing defenses against viral infections (47, 48). It also facilitates the differentiation of monocytes (46) into inflammatory dendritic cells, leading to increased production of cytokines and interferons. Conversely, testosterone suppresses the activity of immune cells and the production of inflammatory cytokines. Thus, compared to men, women exhibit higher humoral and cellular immune responses (49, 50). Next, regarding age, this study found no significant correlation with IgG(S) values, but a significant association was shown with a decrease in T-spot test values. This decline in cellular immune response with age is well-documented (51), and aligns with the concept of immune senescence (48, 52, 53) in older adults (54), a potential factor contributing to decreased antibody production following vaccination in older adults (51, 55), as indicated in previous reports. Additionally, in older women, the biphasic effect of estrogen—immunosuppression at high levels and immunostimulation at low levels (56)—may partially counteract the decline in the adaptive immune response associated with aging (57). Further investigation and consideration of the correlation between IgG(S) and age are needed in future studies.

While this study has explored various factors associated with the occurrence of adverse events, it has not evaluated Adverse Events of Special Interest (AESI). Case reports following vaccination have documented the onset of serious adverse events such as autoimmune myocarditis (58), new autoimmune diseases such as rheumatoid arthritis (59), and conditions like thrombosis and thrombocytopenia (60). Recognizing the risk of such serious adverse events is crucial. On the other hand, it is also important to acknowledge that adverse events, while uncomfortable, may indicate an effective immune response and could serve as a marker for the prevention of serious diseases through vaccination (61) and an effective immune response.

This study had several limitations. First, the participants were recruited through specific networks, which may have introduced a sampling bias, making generalization difficult. Additionally, this study was unable to collect adequate data regarding the severity and duration of adverse events, as well as information on comorbidities. This limitation restricts our ability to comprehensively analyze the overall relationship between systemic adverse events and immune responses following vaccination. Moreover, the accumulation and evaluation of data on AESI were not sufficient. Evaluating AESI is crucial in long-term follow-up studies and actual clinical settings, and remains a challenge for future research. Furthermore, there were missing values in the data (Supplementary Table 1), which could have led to a confounding bias. However, this study is the first within the same cohort to investigate the characteristics and related factors in groups with repeated adverse events.

## 5 Conclusion

In this study, LCA was used to identify two distinct groups based on adverse events following the second-to-fourth COVID-19 vaccinations: one group with fewer adverse events and the other with more frequent adverse events. Age, sex, and a history of allergies were significant factors in the group associated with repeated adverse events. Groups of longitudinal adverse events identified by LCA were expected to be valuable for optimizing vaccination strategies and formulating public health measures.

## Data availability statement

The data analyzed in this study is subject to the following licenses/restrictions: the datasets generated in this study are not publicly available; however, they are available upon reasonable request from the corresponding author. Requests to access these datasets should be directed to MTs, tsubo-m@fmu.ac.jp.

## Ethics statement

The studies involving humans were approved by the Ethics Committees of Hirata Central Hospital (number 2021-0611-1) and Fukushima Medical University (number 2021-116). The studies were conducted in accordance with the local legislation and institutional requirements. The participants provided their written informed consent to participate in this study.

## Author contributions

CY: Data curation, Formal analysis, Writing – original draft, Writing – review & editing. YKo: Data curation, Formal analysis, Writing – original draft. TKa: Investigation, Writing – review & editing. YN: Data curation, Writing – review & editing. HS: Data curation, Writing – review & editing. FO: Data curation, Writing – review & editing. TZ: Data curation, Writing – review & editing. MTa: Data curation, Writing – review & editing. TS: Data curation, Writing – review & editing. AO: Data curation, Writing – review & editing. TA: Data curation, Writing – review & editing. NI: Data curation, Writing – review & editing. YKa: Investigation, Writing – review & editing. AN: Investigation, Writing – review & editing. MW: Investigation, Writing – review & editing. TKo: Investigation, Writing – review & editing. MTs: Data curation, Formal analysis, Funding acquisition, Project administration, Resources, Supervision, Writing – review & editing.

## References

[1] Pal S Shekhar R Kottewar S Upadhyay S Singh M Pathak D. COVID-19 vaccine hesitancy and attitude toward booster doses among US healthcare workers. Vaccines. (2021) 9:1358. doi: 10.3390/vaccines9111358, PMID: 34835289 PMC8617683

[2] Polack FP Thomas SJ Kitchin N Absalon J Gurtman A Lockhart S. Safety and efficacy of the BNT162b2 mRNA COVID-19 vaccine. N Engl J Med. (2020) 383:2603–15. doi: 10.1056/NEJMoa2034577, PMID: 33301246 PMC7745181

[3] Baden LR el Sahly HM Essink B Kotloff K Frey S Novak R. Efficacy and safety of the mRNA-1273 SARS-CoV-2 vaccine. N Engl J Med. (2021) 384:403–16. doi: 10.1056/NEJMoa2035389, PMID: 33378609 PMC7787219

[4] Roy DN Azam MS Islam E. Multi-dimensional potential factors influencing COVID-19 vaccine booster acceptance and hesitancy among university academic community in Bangladesh: a cross-sectional comparative study. PLoS One. (2023) 18:e0281395. doi: 10.1371/journal.pone.0281395, PMID: 37053270 PMC10101431

[5] Chapin-Bardales J Gee J Myers T. Reactogenicity following receipt of mRNA-based COVID-19 vaccines. JAMA. (2021) 325:2201–2. doi: 10.1001/jama.2021.5374, PMID: 33818592

[6] Villanueva P McDonald E Croda J Croda MG Dalcolmo M dos Santos G. Factors influencing adverse events following COVID-19 vaccination. Hum Vaccin Immunother. (2024) 20:2323853. doi: 10.1080/21645515.2024.2323853, PMID: 38445666 PMC10936640

[7] Rutkowski K Mirakian R Till S Rutkowski R Wagner A. Adverse reactions to COVID-19 vaccines: a practical approach. Clin Exp Allergy. (2021) 51:770–7. doi: 10.1111/cea.13880, PMID: 33813758 PMC8250847

[8] MacDonald NE SAGE Working Group on Vaccine Hesitancy. Vaccine hesitancy: definition, scope and determinants. Vaccine. (2015) 33:4161–4. doi: 10.1016/j.vaccine.2015.04.036, PMID: 25896383

[9] Yoshida M Kobashi Y Kawamura T Shimazu Y Nishikawa Y Omata F. Factors associated with COVID-19 vaccine booster Hesitancy: a retrospective cohort study, Fukushima vaccination community survey. Vaccines. (2022) 10:515. doi: 10.3390/vaccines10040515 35455264 PMC9032295

[10] Lazarus JV Ratzan SC Palayew A Gostin LO Larson HJ Rabin K. A global survey of potential acceptance of a COVID-19 vaccine. Nat Med. (2021) 27:225–8. doi: 10.1038/s41591-020-1124-9, PMID: 33082575 PMC7573523

[11] Peters MDJ. Addressing vaccine hesitancy and resistance for COVID-19 vaccines. Int J Nurs Stud. (2022) 131:104241. doi: 10.1016/j.ijnurstu.2022.104241, PMID: 35489108 PMC8972969

[12] Castaldo M Waliszewska-Prosół M Koutsokera M Robotti M Straburzyński M Apostolakopoulou L. Headache onset after vaccination against SARS-CoV-2: a systematic literature review and meta-analysis. J Headache Pain. (2022) 23:41. doi: 10.1186/s10194-022-01400-4, PMID: 35361131 PMC8969402

[13] Hayashi M Morikawa S Goto Y Yoshida T Kimura Y Imaizumi K. Adverse reactions to mRNA coronavirus disease 2019 (COVID-19) vaccine for severe acute respiratory syndrome coronavirus 2 (SARS-CoV-2) in 576 medical staff. Fujita Med J. (2022) 8:79–82. doi: 10.20407/fmj.2021-009, PMID: 35949514 PMC9358674

[14] Menni C Klaser K May A Polidori L Capdevila J Louca P. Vaccine side-effects and SARS-CoV-2 infection after vaccination in users of the COVID symptom study app in the UK: a prospective observational study. Lancet Infect Dis. (2021) 21:939–49. doi: 10.1016/S1473-3099(21)00224-3, PMID: 33930320 PMC8078878

[15] Menni C May A Polidori L Louca P Wolf J Capdevila J. COVID-19 vaccine waning and effectiveness and side-effects of boosters: a prospective community study from the ZOE COVID study. Lancet Infect Dis. (2022) 22:1002–10. doi: 10.1016/S1473-3099(22)00146-3, PMID: 35405090 PMC8993156

[16] Gram MA Nielsen J Schelde AB Nielsen KF Moustsen-Helms IR Sørensen AKB. Vaccine effectiveness against SARS-CoV-2 infection, hospitalization, and death when combining a first dose ChAdOx1 vaccine with a subsequent mRNA vaccine in Denmark: a nationwide population-based cohort study. PLoS Med. (2021) 18:e1003874. doi: 10.1371/journal.pmed.1003874, PMID: 34919548 PMC8726493

[17] di Fusco M Lin J Vaghela S Lingohr-Smith M Nguyen JL Scassellati Sforzolini T. COVID-19 vaccine effectiveness among immunocompromised populations: a targeted literature review of real-world studies. Expert Rev Vaccines. (2022) 21:435–51. doi: 10.1080/14760584.2022.2035222, PMID: 35112973 PMC8862165

[18] Urakawa R Isomura ET Matsunaga K Kubota K. Young age, female sex, and no comorbidities are risk factors for adverse reactions after the third dose of BNT162b2 COVID-19 vaccine against SARS-CoV-2: a prospective cohort study in Japan. Vaccines. (2022) 10:1357. doi: 10.3390/vaccines10081357 36016244 PMC9416095

[19] Kobashi Y Kawamura T Shimazu Y Zhao T Sugiyama A Nakayama A. Humoral immunity after second dose of BNT162b2 vaccine in Japanese communities: an observational cross-sectional study, Fukushima vaccination community survey. Sci Rep. (2022) 12:18929. doi: 10.1038/s41598-022-21797-x, PMID: 36344597 PMC9640658

[20] Yoshida M Kobashi Y Kawamura T Shimazu Y Nishikawa Y Omata F. Association of systemic adverse reaction patterns with long-term dynamics of humoral and cellular immunity after coronavirus disease 2019 third vaccination. Sci Rep. (2023) 13:9264. doi: 10.1038/s41598-023-36429-1, PMID: 37286720 PMC10246541

[21] Kobashi Y Nishikawa Y Kawamura T Kodama T Shimazu Y Obara D. Seroprevalence of SARS-CoV-2 antibodies among hospital staff in rural Central Fukushima, Japan: a historical cohort study. Int Immunopharmacol. (2021) 98:107884. doi: 10.1016/j.intimp.2021.107884, PMID: 34246041 PMC8200307

[22] Yoshida M Kobashi Y Shimazu Y Saito H Yamamoto C Kawamura T. Time course of adverse reactions following BNT162b2 vaccination in healthy and allergic disease individuals aged 5-11 years and comparison with individuals aged 12-15 years: an observational and historical cohort study. Eur J Pediatr. (2023) 182:123–33. doi: 10.1007/s00431-022-04643-0 36224435 PMC9556290

[23] Kobashi Y Shimazu Y Nishikawa Y Kawamura T Kodama T Obara D. The difference between IgM and IgG antibody prevalence in different serological assays for COVID-19; lessons from the examination of healthcare workers. Int Immunopharmacol. (2021) 92:107360. doi: 10.1016/j.intimp.2020.107360, PMID: 33508702 PMC7836839

[24] Kobashi Y Shimazu Y Kawamura T Nishikawa Y Omata F Kaneko Y. Peak IgG antibody titers against SARS-CoV-2 spike protein following immunization with the Pfizer/BioNTech BNT162b2 vaccine. Fukushima J Med Sci. (2022) 68:67–70. doi: 10.5387/fms.2021-28, PMID: 35228456 PMC9071351

[25] Kobashi Y Takebayashi Y Yoshida M Kawamura T Shimazu Y Kaneko Y. Waning of humoral immunity and the influencing factors after BNT162b2 vaccination: a cohort study with a latent growth curve model in Fukushima. Vaccines. (2022) 10:2007. doi: 10.3390/vaccines10122007 36560417 PMC9782062

[26] Tani Y Takita M Kobashi Y Wakui M Zhao T Yamamoto C. Varying cellular immune response against SARS-CoV-2 after the booster vaccination: a cohort study from Fukushima vaccination community survey, Japan. Vaccines. (2023) 11:920. doi: 10.3390/vaccines11050920 37243024 PMC10220831

[27] Saito H Yoshimura H Yoshida M Tani Y Kawashima M Uchiyama T. Antibody profiling of microbial antigens in the blood of COVID-19 mRNA vaccine recipients using microbial protein microarrays. Vaccines. (2023) 11:1694. doi: 10.3390/vaccines11111694 38006026 PMC10674746

[28] Nylund KL Asparouhov T Muthén BO. Deciding on the number of classes in latent class analysis and growth mixture modeling: a Monte Carlo simulation study. Struct Equ Model Multidiscip J. (2007) 14:535–69. doi: 10.1080/10705510701575396

[29] Kaur U Fatima Z Maheshwari K Sahni V Dehade A KL A. Long-term safety analysis of the ChAdOx1-nCoV-19 corona virus vaccine: results from a prospective observational study in priority vaccinated groups in North India. Drug Saf. (2023) 46:553–63. doi: 10.1007/s40264-023-01301-8, PMID: 37133805 PMC10155654

[30] Carfi A Bernabei R Landi F Gemelli Against COVID-19 Post-Acute Care Study Group. Persistent symptoms in patients after acute COVID-19. JAMA. (2020) 324:603–5. doi: 10.1001/jama.2020.12603, PMID: 32644129 PMC7349096

[31] Núñez I Gillard J Fragoso-Saavedra S Feyaerts D Islas-Weinstein L Gallegos-Guzmán AA. Longitudinal clinical phenotyping of post COVID condition in Mexican adults recovering from severe COVID-19: a prospective cohort study. Front Med. (2023) 10:1236702. doi: 10.3389/fmed.2023.1236702, PMID: 37727759 PMC10505811

[32] Huang C Huang L Wang Y Li X Ren L Gu X. 6-month consequences of COVID-19 in patients discharged from hospital: a cohort study. Lancet. (2023) 401:e21–33. doi: 10.1016/S0140-6736(23)00810-3, PMID: 37321233 PMC10258565

[33] Rosenblum HG Gee J Liu R Marquez PL Zhang B Strid P. Safety of mRNA vaccines administered during the initial 6 months of the US COVID-19 vaccination programme: an observational study of reports to the vaccine adverse event reporting system and v-safe. Lancet Infect Dis. (2022) 22:802–12. doi: 10.1016/S1473-3099(22)00054-8, PMID: 35271805 PMC8901181

[34] García-Grimshaw M Ceballos-Liceaga SE Hernández-Vanegas LE Núñez I Hernández-Valdivia N Carrillo-García DA. Neurologic adverse events among 704,003 first-dose recipients of the BNT162b2 mRNA COVID-19 vaccine in Mexico: a nationwide descriptive study. Clin Immunol. (2021) 229:108786. doi: 10.1016/j.clim.2021.108786, PMID: 34147649 PMC8213977

[35] Coggins SA Laing ED Olsen CH Goguet E Moser M Jackson-Thompson BM. Adverse effects and antibody titers in response to the BNT162b2 mRNA COVID-19 vaccine in a prospective study of healthcare workers. Open Forum Infect Dis. (2022) 9:ofab575. doi: 10.1093/ofid/ofab575 35047649 PMC8759445

[36] Green MS Peer V Magid A Hagani N Anis E Nitzan D. Gender differences in adverse events following the Pfizer-BioNTech COVID-19 vaccine. Vaccines. (2022) 10:233. doi: 10.3390/vaccines10020233 35214694 PMC8875740

[37] Sugiyama A Kurisu A Nagashima S Hando K Saipova K Akhmedova S. Seroepidemiological study of factors affecting anti-spike IgG antibody titers after a two-dose mRNA COVID-19 vaccination in 3744 healthy Japanese volunteers. Sci Rep. (2022) 12:16294. doi: 10.1038/s41598-022-20747-x, PMID: 36175506 PMC9520958

[38] Haider SMS Alvi SA Khan H Majeed R Syed T Anwar A. Common side effects of Pfizer COVID-19 vaccine: an experience from Pakistan. Cureus. (2023) 15:e40878. doi: 10.7759/cureus.40878, PMID: 37492805 PMC10363686

[39] Inoue S Igarashi A Morikane K Hachiya O Watanabe M Kakehata S. Adverse reactions to BNT162b2 mRNA COVID-19 vaccine in medical staff with a history of allergy. Respir Investig. (2022) 60:248–55. doi: 10.1016/j.resinv.2021.11.007, PMID: 34920980 PMC8648579

[40] Kaur U K LA Chauhan M Joshi A Das A Kansal S. A prospective observational study on BBV152 coronavirus vaccine use in adolescents and comparison with adults: interim results of the first real-world safety analysis. Drug Saf. (2022) 45:1099–109. doi: 10.1007/s40264-022-01226-8, PMID: 36030299 PMC9419918

[41] Kaur U Ojha B Pathak BK Singh A Giri KR Singh A. A prospective observational safety study on ChAdOx1 nCoV-19 corona virus vaccine (recombinant) use in healthcare workers-first results from India. EClinicalMedicine. (2021) 38:101038. doi: 10.1016/j.eclinm.2021.101038, PMID: 34505032 PMC8413251

[42] Takahashi W Mizuno T Hara K Ara Y Hurutani R Agatsuma T. Association of systemic adverse reactions and serum SARS-CoV-2 spike protein antibody levels after administration of BNT162b2 mRNA COVID-19 vaccine. Intern Med. (2022) 61:3205–10. doi: 10.2169/internalmedicine.9699-22, PMID: 35989281 PMC9683821

[43] Yoshifuji A Toda M Ryuzaki M Oyama E Kikuchi K Kawai T. T-cell response and antibody production induced by the COVID-19 booster vaccine in Japanese chronic kidney disease patients treated with hemodialysis. Vaccines. (2023) 11:653. doi: 10.3390/vaccines11030653 36992238 PMC10057502

[44] Roved J Westerdahl H Hasselquist D. Sex differences in immune responses: hormonal effects, antagonistic selection, and evolutionary consequences. Horm Behav. (2017) 88:95–105. doi: 10.1016/j.yhbeh.2016.11.017, PMID: 27956226

[45] Bhatia A Sekhon HK Kaur G. Sex hormones and immune dimorphism. Sci World J. (2014) 2014:159150. doi: 10.1155/2014/159150 PMC4251360 25478584

[46] Klein SL Flanagan KL. Sex differences in immune responses. Nat Rev Immunol. (2016) 16:626–38. doi: 10.1038/nri.2016.90 27546235

[47] Robinson DP Hall OJ Nilles TL Bream JH Klein SL. 17beta-estradiol protects females against influenza by recruiting neutrophils and increasing virus-specific CD8 T cell responses in the lungs. J Virol. (2014) 88:4711–20. doi: 10.1128/JVI.02081-13, PMID: 24522912 PMC3993800

[48] Cannizzo ES Clement CC Sahu R Follo C Santambrogio L. Oxidative stress, inflamm-aging and immunosenescence. J Proteome. (2011) 74:2313–23. doi: 10.1016/j.jprot.2011.06.005, PMID: 21718814

[49] Klein SL Jedlicka A Pekosz A. The Xs and Y of immune responses to viral vaccines. Lancet Infect Dis. (2010) 10:338–49. doi: 10.1016/S1473-3099(10)70049-9, PMID: 20417416 PMC6467501

[50] Fish EN. The X-files in immunity: sex-based differences predispose immune responses. Nat Rev Immunol. (2008) 8:737–44. doi: 10.1038/nri2394, PMID: 18728636 PMC7097214

[51] Romero-Olmedo AJ Schulz AR Hochstätter S das Gupta D Virta I Hirseland H. Induction of robust cellular and humoral immunity against SARS-CoV-2 after a third dose of BNT162b2 vaccine in previously unresponsive older adults. Nat Microbiol. (2022) 7:195–9. doi: 10.1038/s41564-021-01046-z, PMID: 35013593

[52] Fink AL Klein SL. Sex and gender impact immune responses to vaccines among the elderly. Physiology. (2015) 30:408–16. doi: 10.1152/physiol.00035.2015, PMID: 26525340 PMC4630198

[53] Castelo-Branco C Soveral I. The immune system and aging: a review. Gynecol Endocrinol. (2014) 30:16–22. doi: 10.3109/09513590.2013.852531 24219599

[54] Walsh EE Frenck RW Jr Falsey AR Kitchin N Absalon J Gurtman A. Safety and immunogenicity of two RNA-based COVID-19 vaccine candidates. N Engl J Med. (2020) 383:2439–50. doi: 10.1056/NEJMoa2027906, PMID: 33053279 PMC7583697

[55] Müller L Andrée M Moskorz W Drexler I Walotka L Grothmann R. Age-dependent immune response to the Biontech/Pfizer BNT162b2 coronavirus disease 2019 vaccination. Clin Infect Dis. (2021) 73:2065–72. doi: 10.1093/cid/ciab381, PMID: 33906236 PMC8135422

[56] Channappanavar R Fett C Mack M Ten Eyck PP Meyerholz DK Perlman S. Sex-based differences in susceptibility to severe acute respiratory syndrome coronavirus infection. J Immunol. (2017) 198:4046–53. doi: 10.4049/jimmunol.1601896, PMID: 28373583 PMC5450662

[57] Márquez EJ Chung CH Marches R Rossi RJ Nehar-Belaid D Eroglu A. Sexual-dimorphism in human immune system aging. Nat Commun. (2020) 11:751. doi: 10.1038/s41467-020-14396-9, PMID: 32029736 PMC7005316

[58] Singh R Chakrabarti SS Gambhir IS Verma A Kumar I Ghosh S. Acute cardiac events after ChAdOx1 nCoV-19 corona virus vaccine: report of three cases. Am J Ther. (2022) 29:e579–85. doi: 10.1097/MJT.0000000000001472, PMID: 35175717

[59] Singh R Kaur U Singh A Chakrabarti SS. Refractory hypereosinophilia associated with newly diagnosed rheumatoid arthritis following inactivated BBV152 COVID-19 vaccine. J Med Virol. (2022) 94:3482–7. doi: 10.1002/jmv.27742, PMID: 35352366 PMC9088458

[60] Schultz NH Sørvoll IH Michelsen AE Munthe LA Lund-Johansen F Ahlen MT. Thrombosis and thrombocytopenia after ChAdOx1 nCoV-19 vaccination. N Engl J Med. (2021) 384:2124–30. doi: 10.1056/NEJMoa2104882, PMID: 33835768 PMC8112568

[61] Jangra S Ye C Rathnasinghe R Stadlbauer D PVI study group Krammer F. The E484K mutation in the SARS-CoV-2 spike protein reduces but does not abolish neutralizing activity of human convalescent and post-vaccination sera. medRxiv. (2021). doi: 10.1101/2021.01.26.21250543

