# Supplementary material for: Group of longitudinal adverse event patterns after the fourth dose of COVID-19 vaccination with a latent class analysis
Source: Front Public Health. 2024 Jul 30;12:1406315. doi: 10.3389/fpubh.2024.1406315 (PMC11320210; doi:10.3389/fpubh.2024.1406315)
Supplement: Supplementary file 1 [file Table_1.docx]

Supplementary Material

**Supplementary Table1. Summary of the number of missing data each category**

|  | Group 1  (Low adverse reaction group) | Group 2  (High adverse reaction group) |
| --- | --- | --- |
| Sex | 25 | 108 |
| Smoking habit | 13 | 7 |
| Alcohol consumption | 15 | 8 |
| Daily medicine |  |  |
| Steroid | 10 | 11 |
| Immunosuppression | 11 | 10 |
| Biologics | 13 | 11 |
| Comorbidity |  |  |
| Hypertension | 1 | 0 |
| Diabetes | 1 | 0 |
| Dyslipidemia | 1 | 0 |
